# Network pharmacology-based strategy combined with molecular docking to explore the potential mechanism of agarwood against recurrent aphthous stomatitis

**DOI:** 10.1097/MD.0000000000037660

**Published:** 2024-03-29

**Authors:** Si-Yu Tao, Hai-Ou Zhang, Qing Yuan, Chu-Xing Li, Xiang Guo, Diwas Sunchuri, Zhu-Ling Guo

**Affiliations:** a School of Dentistry, Hainan Medical University, Haikou, P.R. China; b Department of Dentistry, The First Affiliated Hospital of Hainan Medical University, Haikou, P.R. China; c The 928th Hospital of People’s Liberation Army Joint Logistic Support Force, Haikou, P.R. China; d Department of Dentistry, The Second Affiliated Hospital of Hainan Medical University, Haikou, P.R. China; e School of International Education, Hainan Medical University, Haikou, P.R. China; f Department of Health Management Center, The First Affiliated Hospital of Hainan Medical University, Haikou, P.R. China.

**Keywords:** agarwood, KEGG pathways, network pharmacological analysis, protein-protein interaction (PPI) network, recurrent aphthous stomatitis, target genes

## Abstract

To explore the antiinflammatory mechanism of agarwood on recurrent aphthous stomatitis (RAS). RAS is the most common mucosal disease in the oral cavity. The clinical application of traditional Chinese medicine found that agarwood has significant curative effect on peptic ulcer, but the effect and mechanism of agarwood on RAS remain unclear. This study is intended to predict the potential antiinflammatory mechanisms by which agarwood acts on RAS through network pharmacology and molecular docking. TCMSP database was used to screen the active components of agarwood. RAS targets were screened in Genecards, DisGeNET, and OMIM database. Venny, an online tool, screens for interacting genes between the two. Cytoscape software was used to construct the gene regulation map of active compounds target of agarwood. String Database building protein-protein interaction network. Gene ontology and Kyoto Encyclopedia of Genes and Genomes pathways were enriched in DAVID database. The key active ingredients and core targets were further verified by molecular docking. There were 9 effective compounds and 186 target genes in agarwood; RAS has 793 target genes. There were 41 interacting genes between agarwood and RAS. Interleukin 6, tumor necrosis factor, interleukin 1 beta, and cellular component motif ligand 2 may be key targets. Gene ontology and Kyoto Encyclopedia of Genes and Genomes enrichment analyses predicted multiple pathways associated with RAS. Molecular docking results showed that the active compounds of agarwood combined well and stably with the target. The Chinese herbal medicine agarwood can relieve the inflammation of RAS through multiple targets and various ways. Its active compounds may be nominated as candidates for antiinflammatory drugs of RAS.

## 1. Introduction

Recurrent aphthous stomatitis (RAS) is commonly occurring mucosal disease in the mouth.^[1]^ The usual clinical symptoms are a yellow-gray pseudomembrane covering the necrotic center of the ulcer, surrounded by red congestion, a sunken center of necrotic ulcer, and a painful round ulcer surface.^[2,3]^ RAS is more common in areas with poor keratinization, such as the cheek and tongue.^[4]^ RAS is recurrent and self-limiting. Pain is often the main complaint of patients with RAS. The etiology of RAS is complex, and many clinical and animal experiments have shown that the occurrence of RAS may be closely related to infection, immune dysfunction, mechanical trauma, and mental factors.^[5]^ There is no specific drug to cure RAS. Clinically, the drugs commonly used to relieve RAS are immunosuppressive drugs, which can affect the immune function of the body after long-term use. Traditional Chinese medicine has lesser side effects and more gentle in action.

Agarwood is called as *chen xiang* in Chinese. Agarwood is mostly found in China, India, and Southeast Asian countries.^[6]^ In ancient times, agarwood was often used as traditional Chinese medicine or as an auxiliary material to treat various diseases.^[7]^ Modern medicine further confirmed that agarwood contains some active substances with analgesic, sedative, antibacterial, and antiinflammatory effects.^[8]^ Agarwood, as a traditional spice and precious medicine, enjoys a high reputation in the world. Agarwood essential oil from agarwood has been gradually used in clinical treatment of various diseases.^[9,10]^ The clinical application of traditional Chinese medicine found that agarwood has significant curative effect on peptic ulcer, but the effect and mechanism of agarwood on RAS remain unclear.^[11]^

This study was carried out to predict the active compounds and core targets of agarwood and their antiinflammatory effects on RAS.

## 2. Materials and methods

For this bioinformatics data analysis, approval by the ethics committee was not necessary.

### 2.1. Screening of active compounds and target of agarwood

The active compounds of agarwood were obtained from TCM Systematic Pharmacology Database (TCMSP, https://tcmsp-e.com/). TCMSP is a unique herbal medicine system pharmacological platform where we can get the relationship between drugs, targets, and diseases. Oral bioavailability and drug likeness were screened through the TCMSP database using absorption, distribution, metabolism, and excretion criteria. In this study, oral bioavailability ≥30% and drug likeness ≥0.18 were set as the criteria to screen the active compounds of agarwood identified in TCMSP database. The active compounds and corresponding targets of agarwood were extracted from TCMSP. The obtained targets were calibrated by Uniprot (https://www.uniprot.org/) data, nonhuman genes were deleted, and duplicate data were removed to obtain standardized gene names. Based on screening out of agarwood active compounds and targets, using Cytoscape 3.8.1 software (http://www.cytoscape.org/) to build “drug-compounds-target” network.

### 2.2. Screening of targets associated with RAS

By using GeneCards (https://www.genecards.org/), Disgenet (https://www.disgenet.org/), and OMIM (https://omim.org/), keywords “recurrent aphthous stomatitis” were input in the database and all related target genes were obtained. All the targets of the 3 databases were integrated into Excel, duplicate genes were removed, and the gene information of RAS target was finally obtained after correction by Uniprot (https://www.uniprot.org/) database.

### 2.3. Prediction of RAS target by action of agarwood

Screening of agarwood targets and targets of the RAS were uploaded to Venny 2.1 online mapping tools (https://bioinfogp.cnb.csic.es/tools/venny/index.html), and then the Venn diagram was exported. Finally, the intersection target of the 2 was used as a potential target for the treatment of RAS by agarwood.

### 2.4. Construction of target protein interaction network

The interaction gene was imported into the String database (https://string-db.org/). The “species” was “*Homo sapiens*,” and the threshold for minimum interaction “medium confidence” was set as 0.4. The results are stored in TSV format, the TSV file is imported into Cytoscape3.8.1, and the network is analyzed. After obtaining (protein-protein interaction, PPI) network of protein interactions, core targets were calculated using CytoHubba plug-in of Cytoscape.

### 2.5. Functional analysis

The drug-disease interaction gene were uploaded to DAVID database (https://david.ncifcrf.gov/) for interactive genetic gene ontology (GO) and Kyoto Encyclopedia of Genes and Genomes (KEGG) pathways enrichment analysis. GO categories: biological processes (BP), cellular components (CC), and molecular functions (MF). The top 10 BP, CC, MF in GO function and 20 pathways in KEGG pathways related to RAS (*P* < .01) were selected as the main gene function enrichment processes and signaling pathways for drug treatment of disease, and the mechanism of action of drug treatment of RAS was predicted.

### 2.6. Molecular docking verification

The core components were docked with the core targets in a molecular level, and the structures of major compounds with high degree were obtained from the pubchem (https://pubchem.ncBI.nlm.nih.gov) database. The 3D structures of the top 4 core targets with *P* values were retrieved from the PDB (http://www.rcsb.org/) database. The 3D structures were dehydrated, hydrogenated, and charged by Auto Dock Tools 1.5.6, and saved in pdbqt format. In the same way, the mol2 structure of active compounds was converted into pdbqt format. Autodock Vina was used to dock the target with the active components of agarwood, and affinity value (unit: kcal/mol) was used to evaluate the binding activity. Finally, Pymol 2.4.1 was used for visualization.

## 3. Results

### 3.1. Screening of target of active compounds

A total of 9 effective compounds were screened in TCMSP database (Table 1). A total of 186 target genes were screened out by deleting duplicate targets and nonhuman genes.

**Table 1:** Parameters of active compounds of agarwood.

Mol. ID	Molecule name	OB (%)	DL
MOL010916	Nubigenol	42.55	0.19
MOL010913	C09495	77.09	0.25
MOL000098	Quercetin	46.43	0.28
MOL010495	6,7-Dimethoxy-2-(2-phenylethyl)chromone	31.93	0.3
MOL010496	DMPEC	32.38	0.39
MOL010907	Norboldine	40.92	0.46
MOL010917	Boldine	31.18	0.51
MOL000359	Sitosterol	36.91	0.75
MOL000358	β-Sitosterol	36.91	0.75

OB = oral bioavailability, DL = drug likeness.

### 3.2. The construction of the “compound interacting gene” network of agarwood

**Figure 1.**
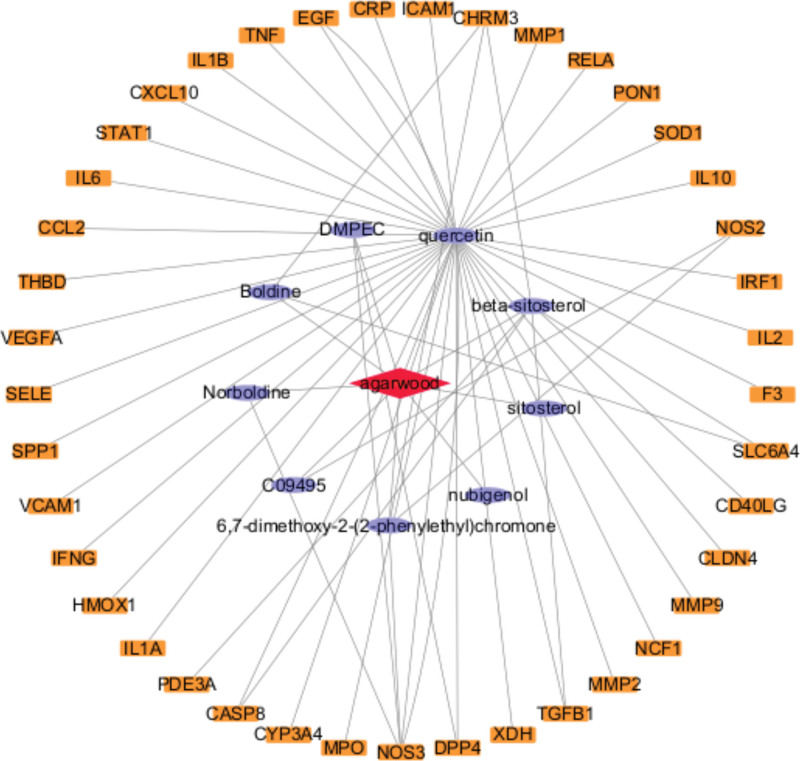
Network diagram of “Agarwood Compound-Interactive Gene.” The purple diamond nodes inside represents the active ingredient of agarwood, the yellow rectangle on the outside represent related target genes.

Cytoscape software was used to map the “compound-interacting gene” network (Fig. 1). It can be seen from the network diagram that there is a one to many relationship between the active components of agarwood and the interactive genes. The diagram contains 51 nodes and 62 edges. The component with a higher degree in the figure is qucertin and β-sitosterol, suggesting that these compounds may play crucial role in the treatment of RAS with agarwood.

### 3.3. Screening of target of RAS

The related targets of RAS were obtained in GeneCards, Disgenet, and OMIM database, and then the duplicated data were removed to obtain 793 disease targets of RAS.

### 3.4. Venny analysis

**Figure 2.**
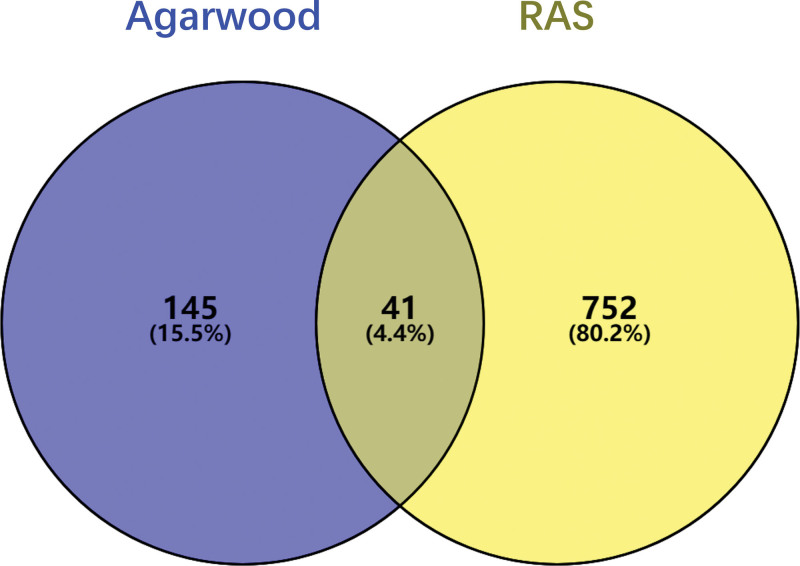
Venn diagram of intersecting targets of agarwood and RAS. The blue circle indicates the target of agarwood, the yellow circle indicates the target of RAS screened from the database, and the crossed dark gray part in the middle indicates the target of agarwood that may act on the RAS. RAS = recurrent aphthous stomatitis.

The above 9 kinds of active ingredient-related targets (186 targets) and RAU-related targets (793 targets) were imported into the online website VENNY 2.1.0 and Venn diagram was drawn. The results showed that there were 41 interacting genes between active components of agarwood and RAS. These 41 interacting genes may be potential target genes for the effect of agarwood on RAS (Fig. 2).

### 3.5. PPI network analysis

**Figure 3.**
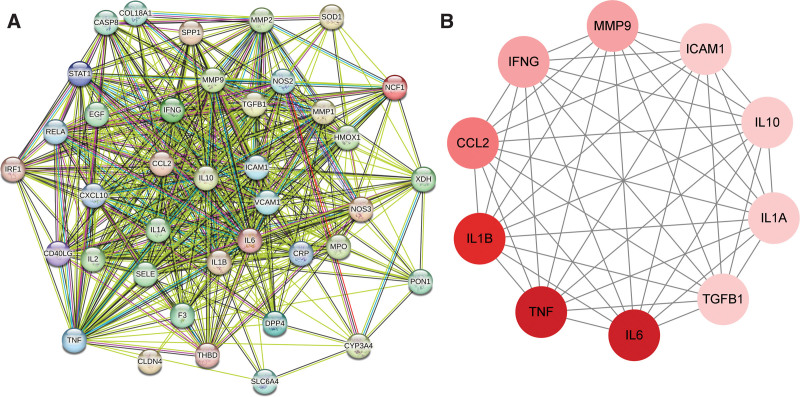
The interaction network of agarwood and RAS targets and key target proteins. (A) The network of agarwood and RAS target; (B) The interaction network of the key target proteins of agarwood.

Forty-one potential targets of agarwood acting on RAS were imported into string, unconnected targets were removed, network files were saved as TSV format and imported into Cytoscape 3.8.1 software to map the protein interaction network (Fig. 3A). Furthermore, the cytoHubba module was used to calculate the degree of each node and then the core target interaction network was built. The top 10 genes were interleukin 6 (IL6), tumor necrosis factor (TNF), interleukin 1 beta (IL1B), CC motif ligand 2 (CCL2), interferon gamma, matrix metallopeptidase 9, intercellular adhesion molecule 1, interleukin 10, interleukin 1 alpha, and transforming growth factor beta 1, which were identified as the hub genes (Fig. 3B).

### 3.6. GO and KEGG enrichment analysis

**Figure 4.**
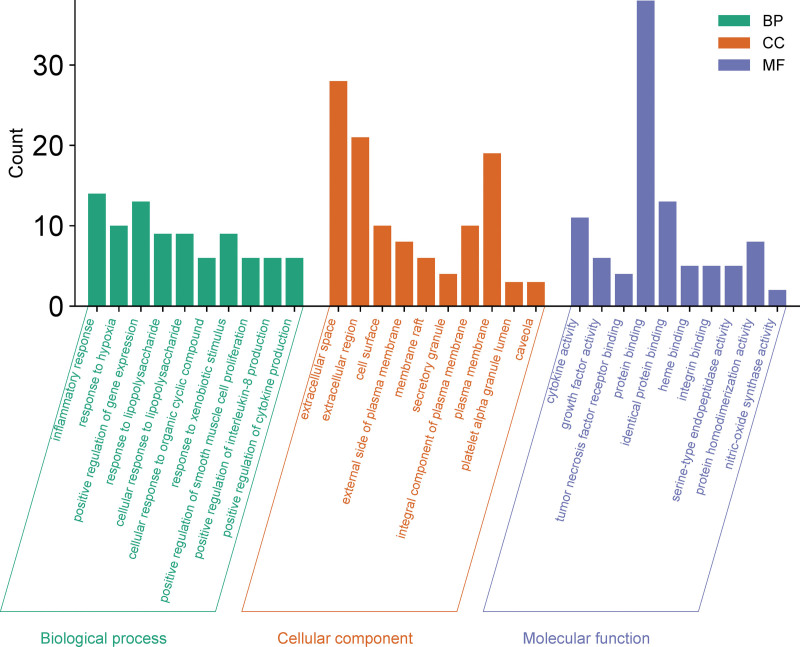
Gene ontology terms of candidate targets of agarwood against RAS. The top 10 GO functional categories with *P* < .05 were selected. The green bars represent biological processes; orange bars represent cellular component; blue bars represent molecular function. GO = gene ontology, RAS = recurrent aphthous stomatitis.

GO and KEGG enrichment analysis was performed for 41 interacting genes in DAVID database. A total of 336 GO items were selected, with *P* < .01 as the criterion. There were 285 BP items which were significantly enriched in the treatment of RAS by agarwood. inflammatory response is the main BP. There are 19 CC-related items, and CC is dominated by the extracellular space. There are 32 items related to MF, and cytokine activity is the main MF (Fig. 4). Export and visualize the top 10 BP, CC, and MF items.

**Figure 5.**
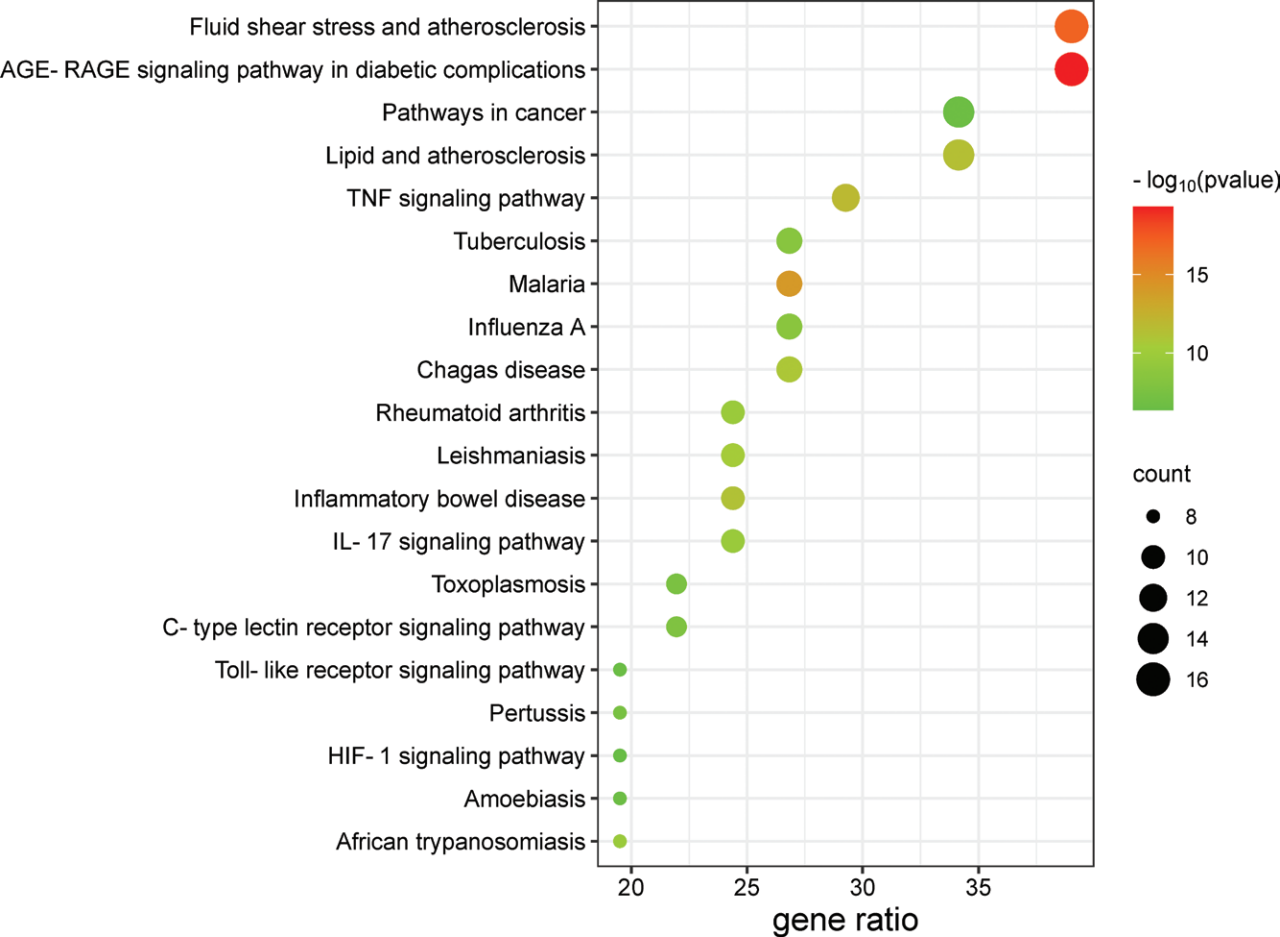
Bubble diagram of pathway enrichment analysis of target genes of agarwood against RAS. The number of genes enriched in each KEGG pathway item is represented by the size of a circle and the *P* value is represented by a different color. RAS = recurrent aphthous stomatitis.

**Figure 6.**
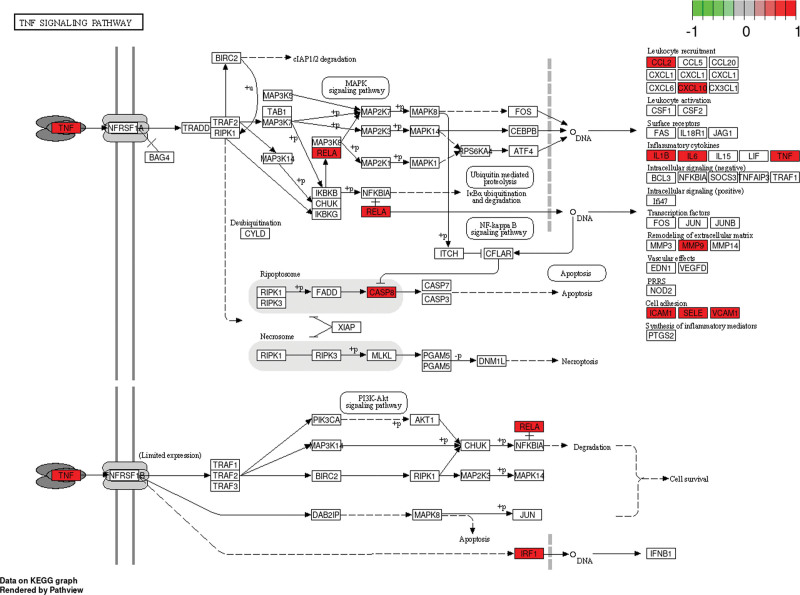
The mechanism through which agarwood against RAS. Red rectangle are the interaction targets of agarwood and RAS contained in the TNF signaling pathway. RAS = recurrent aphthous stomatitis, TNF = tumor necrosis factor.

The top 20 KEGG pathways will be filtered according to the *P* value, and the bubble map will be drawn according to the *P* value (Fig. 5). The results showed that the TNF pathway was enriched in large amounts (Fig. 6).

### 3.7. Molecular docking

**Figure 7.**
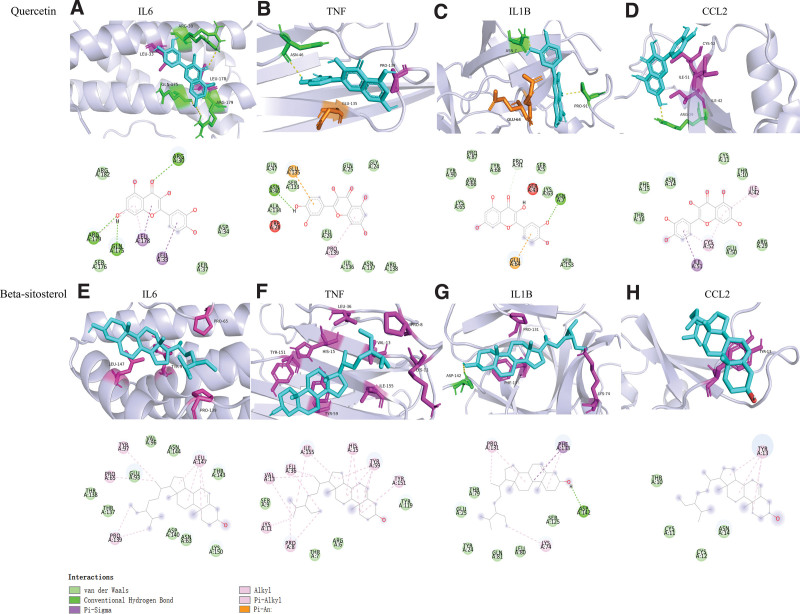
Molecular docking between the main active components of agarwood and core targets. (A–D) 3D and 2D images of the docking of quercetin with IL6, TNF, IL1B, and CCL2. (A) quercetin binds to the active site of IL6 stably through ARG-30, LEU-33, GLN-175, LEU-178, and ARG-179 on IL6 target proteins; (B) quercetin binds stably to the active site of TNF via ASN-46, PRO-139, and GLU-135 on TNF target proteins; (C) quercetin binds stably to the IL1B active site via ASN-7, PRO-91, and GLU-64 on IL1B target proteins; (D) quercetin binds stably to the active site of CCL2 through CYS-52, ILE-51, ILE-42, and ARG-29 on the target protein of CCL2. (E–H) 3D and 2D images of the docking of β-sitosterol with IL6, TNF, IL1B, and CCL2; (E) β-sitosterol binds to the active site of IL6 stably through LEU-147, TYR-97, PRO-65, and PRO-13 on IL6 target proteins; (F) β-sitosterol binds to the active site of TNF stably through TYR-151, TYR-59, HIS-15, LEU-36, ILE-155, LYS-11, VAL-13, and PRO-8 on the TNF target protein; (G) β-sitosterol binds to the IL1B active site stably through the ASP-142, PH-133, PRO-131, and LYS-74 of IL1B target proteins; (H) β-sitosterol binds stably to the active site of CCL2 via TYR-13 on the CCL2 target protein.

Quercetin and β-sitosterol, the core components with the highest concentration of agarwood, were docked in a molecular level with IL6 (PDB: 1ALU), TNF (PDB: 1TNF), IL1β (PDB: 1I1B), CCL2 (PDB: 1DOK), the top 4 key targets in the PPI network. The docking results showed that both quercetin and β-sitosterol could be combined into the docking bag. At the same time, the above target genes IL6, TNF, IL1, and CCL2 had low affinity to the main components of agarwood. Affinity <–5.0 (kcal/mol) indicates that the active compound has a good docking result with the target.^[12]^ The smaller the Affinity value, the stronger the binding force, indicating that the target proteins have good docking activity (Table 2, Fig. 7).

**Table 2:** The docking results of quercetin and β-sitosterol with key gene molecules.

Compound	Target	PDB ID	Energy	Amino acids
Quercetin	IL6	1ALU	-7.1	ARG-30, LEU-33, GLN-175, LEU-178, ARG-179
	TNF	1TNF	-6.8	ASN-46, PRO-139, GLU-135
	IL1β	1I1B	-7.6	ASN-7, PRO-91, GLU-64
	CCL2	1DOK	-6.4	CYS-52, ILE-51, ILE-42, ARG-29
β-Sitosterol	IL6	1ALU	-6.7	LEU-147, TYR-97, PRO-65, PRO-13
	TNF	1TNF	-6.8	TYR-151, TYR-59, HIS-15, LEU-36, ILE-155, LYS-11, VAL-13, PRO-8
	IL1β	1I1B	-7.4	ASP-142, PHE-133, PRO-131, LYS-74
	CCL2	1DOK	-6.7	TYR-13

## 4. Discussion

RAS are recurrent and self-limiting, but they often affect eating due to pain.^[13,14]^ The treatment of RAS usually has 3 purposes: to reduce pain and inflammation, to prevent secondary infections, and to reduce the duration and recurrence of ulcers.^[15]^ However, the clinical application of drugs such as immunosuppressant for relieving RAS is often limited by many side effects.^[16]^ Agarwood, as a traditional Chinese medicine, has the advantages of less side effects and alleviating inflammation. It is promising to replace traditional antiinflammatory drugs to relieve RAS. However, evidence-based studies are still lacking in this field.

In this study, we studied the possible mechanism of the effect of agarwood on RAS by using network pharmacology. Network pharmacology is a combination of clustering algorithms and network topology, and it is a discipline that studies the interaction between drugs and organisms using network analysis, systems biology, and pharmacology. By constructing and analyzing drug-target-disease and other multi-level biological networks, it explores the relationship between compounds and disease target genes, and then reveals the mechanism of action, target, and efficacy of drugs. Therefore, we screened the central compounds, the most potential gene targets, and pathways of agarwood through network pharmacology to provide a more accurate direction for the study of its antiinflammatory mechanism in RAS.

After further study of agarwood, we found 9 active ingredients (quercetin, β-sitosterol, 6,7-dimethoxy-2-(2-phenylethyl)chromone, nubigenol, C09495, DMPEC, norboldine, boldine, and sitosterol) may play a key role in the treatment of RAS. Quercetin and β-sitosterol compounds of agarwood with higher degree were screened by network pharmacology. Quercetin is a flavonoid found in fruits and vegetables. Recently, it has been reported that quercetin is able to promote wound healing process.^[17–20]^ The pathogenic factors of oral ulcer are related to flora disorder, anxiety, and inflammation. β-Sitosterol is a natural phytosterol that is one of the most abundant in plants, which has anticancer, antiinflammatory, antibacterial, and antianxiety effects. Studies have shown that β-sitosterol can reduce the serum levels of inflammatory markers (IL-1β and iNOS) in rats.

In addition, PPI network suggested that multiple targets, such as IL6, TNF, IL1B, CCL2, interferon gamma, matrix metallopeptidase 9, intercellular adhesion molecule 1, interleukin 10, interleukin 1 alpha, and transforming growth factor beta 1, played crucial role in the process of the effect of agarwood on RAS. IL6, produced by activated T cells, is a pleiotropic cell signaling molecule that participates in many physiological processes and is closely related to the occurrence and development of many diseases.^[21]^ IL6 is secreted mainly by mononuclear macrophages in the periphery and can induce acute response to infection or injury. Systematic review and meta-analysis have concluded that the level of IL6 in saliva of patients with oral ulcers is significantly higher than that of normal controls.^[22]^ TNF is a small protein secreted by macrophages. TNFα is a useful diagnostic marker for RAS. In healthy individuals, TNFα is significantly lower than RAS.^[23]^ The pathogenesis of RAS is associated with an increase in the pro-inflammatory cytokine TNFα. This cytokine plays an important role in the development of ulcerative lesions. IL-1β is a pro-inflammatory cytokine produced by innate immune system cells.^[24]^ The association between interleukin family gene polymorphisms and RAS risk was determined by meta-analysis, and IL-1β + 3954C/T polymorphisms were found to be associated with RAS susceptibility.^[25]^ The CCL2 promotes the migration of inflammatory cells through chemotactic action and the activation of integrin, and participates in the occurrence and maintenance of inflammatory response in the body.^[26]^When inflammation occurs in the body, CCL2 quickly and efficiently recruits immune cells to the site of inflammation.^[27]^ CCL2 is a key regulator and therapeutic target for oral periodontitis.^[28]^

The treatment of RAS by agarwood involves several BPs, and BP mainly focuses on the inflammatory response. This finding suggests that agarwood may act on RAS by regulating pathways associated with the inflammatory response. In addition, agarwood acts on RAS involving multiple signaling pathways, with TNF signaling pathway ranking high. TNF plays a regulatory role in reducing, maintaining, and improving inflammatory responses.

The results of molecular docking showed that quercetin and kaempferol, the top 2 active compounds in the “compound target” network, had good binding activity with IL6, TNF, IL1B, and CCL2. This suggests that the pharmacodynamic mechanism of agarwood therapy for RAS has sufficient material basis.

It has been confirmed that the secondary products of agarwood have antiinflammatory properties, especially on the digestive system. Agarwood alcohol extract may prevent stomach ulcers by inhibiting oxidative stress and inflammation. It has been found that pretreatment of gastric tissue with agarwood alcohol extracts can significantly reduce ethanol-induced gastric ulcer index and ulcer area.^[11]^ Oral and gastrointestinal tract belong to the same digestive tract, and there are similarities and correlation in histology and pathogenesis. Agarwood leaves have long been used in Southeast Asia to treat skin diseases. Therefore, combined with the prediction of our network pharmacology and previous experimental results, agarwood may be able to slow the occurrence and development of RAS.

But there are still limitations to the study. Network pharmacology can only report plants and components that have been discovered.^[29]^ Therefore, we cannot search the database for components of agarwood that have not yet been discovered. In the future, more basic experiments and clinical studies of agarwood are still needed to enrich the conclusions of this paper.

## 5. Conclusion

This study revealed the underlying efficacy and mechanisms of multi-target and multi-pathway of agarwood in RAS treatment. Our results suggest that agarwood is a promising candidate drug for RAS treatment.

## Author contributions

**Conceptualization:** Zhu-Ling Guo, Si-Yu Tao.

**Investigation:** Si-Yu Tao, Hai-Ou Zhang.

**Methodology:** Si-Yu Tao, Hai-Ou Zhang, Qing Yuan, Chu-Xing Li, Xiang Guo.

**Project administration:** Si-Yu Tao, Qing Yuan, Chu-Xing Li.

**Resources:** Si-Yu Tao, Qing Yuan, Xiang Guo.

**Software:** Si-Yu Tao, Chu-Xing Li.

**Supervision:** Zhu-Ling Guo, Hai-Ou Zhang.

**Validation:** Zhu-Ling Guo, Diwas Sunchuri.
